# Abnormal blood concentration changes in a 71-year-old female who survived a 10,000mg overdose of clozapine: a case report

**DOI:** 10.1186/s12888-024-05582-w

**Published:** 2024-02-14

**Authors:** Yonghua Wu, Ziyan Zhou, Ziyi Ai, Tiancheng Wang, Liyan Cui

**Affiliations:** Department of Laboratory Medicine, Peking University Third Hospital, No.49, North Garden Rd, Haidian District, Beijing, 100191 China

**Keywords:** Clozapine, Therapeutic drug monitoring, Drug overdose, Liquid chromatography-mass spectrometry

## Abstract

**Background:**

Clozapine is a highly effective second-generation antipsychotic with few extrapyramidal reactions, making it a preferred choice among clinicians. However, instances of acute clozapine poisoning resulting from suicide attempts and misuse have been reported. Through our review of existing literature, we identified that we believe to be the highest recorded overdose of clozapine in elderly patients, resulting in a nonfatal outcome.

**Case presentation:**

The case report involves a 71-year-old female with a history of depression who ingested a dose of 10,000 mg of clozapine. Approximately 6 h after the overdose, the clozapine level was 5,200 μg/L, significantly surpassing the recommended therapeutic concentration range of 350–600 μg/L. After gastric lavage and hemoperfusion, the blood level dropped to 1847.11 μg/L. Notably, during therapeutic drugs monitoring (TDM), we found a perplexing spike in the patient’s blood level to 5554.15 μg/L after the second hemoperfusion.

**Conclusion:**

In this case we mainly focused on the abnormal fluctuations in the concentration of clozapine. We conducted a comprehensive analysis of potential factors contributing to this abnormal phenomenon in terms of the patient’s age, clinical symptoms, various laboratory test indexes, and the pharmacokinetics of clozapine. Our findings underscore the importance of timely TDM and the precision of results in managing elderly patients experiencing high-dose clozapine poisoning.

## Background

Clozapine, one of the dibenzodiazepine drugs, is commonly used in antipsychotic treatment. In contrast to typical antipsychotics, it does not produce significant extrapyramidal side effects. Clozapine has been utilized as an antipsychotic drug due to its simultaneous affinity for both dopamine and serotonin receptors. It can improve the positive symptoms of schizophrenia patients by blocking the dopamine 4 receptor(DA4R) on the mesolimbic dopamine pathway and promote the release of dopamine by blocking the 5-hydroxy tryptamine receptor in the brain, and then reduce its negative symptoms [1].

Clozapine is well absorbed after oral administration which does not affect its bioavailability. However, fixed oral doses of clozapine can produce up to 45-fold inter-individual variability in blood concentration [2]. Therefore, therapeutic drug monitoring (TDM) is necessary for safe and effective medication administration and treatment of clozapine overdose [3]. According to the 2017 AGNP guidelines, the recommended therapeutic drug concentration range for clozapine is 350–600 μg/L, and the laboratory alert concentration is 1000 μg/L, which increases the risk of drug intoxication. The laboratory must give feedback immediately to the prescribing physician [4]. Generally, the blood concentration of patients with clozapine overdose will gradually decrease after hemoperfusion [5]. However, several factors may affect the clozapine blood concentration, such as sex, age, dose, concomitant medications, and smoking history [6]. Many case reports have linked infection with a blood clozapine concentration increase [7]. Compared to other populations, recent research suggests that individuals of Asian descent tend to exhibit lower metabolism of clozapine, as well as higher clozapine concentration-to-dose ratios (C/D), indicating lower clearance is significantly associated with blood clozapine concentration increase [8].

Based on the literature we have retrieved, massive-dose clozapine administration in elderly females (> 70 years) have rarely been reported. In the current study, we report a case of abnormal blood concentration changes in a 71-year-old female who survived a 10,000 mg overdose of clozapine and tried to explore the possible causes.

## Case presentation

A 71-year-old female, diagnosed with depression 20 years ago, was admitted to the emergency room on this occasion with the primary symptoms of nausea, vomiting, and impaired consciousness for 6 h after taking 400 clozapine tablets (25 mg/tablet). The patient had a history of diabetes mellitus, hypertension, coronary artery disease, and post-stenting. The examination showed a drowsy state, hypotension, muscle tremor, and rapid heart rate. As medical technologists, we used liquid chromatography-mass spectrometry technology to detect blood clozapine concentration, the initial concentration was 5200 μg/L, nearly nine times above the upper limit of the therapeutic concentration reference range. She was diagnosed with a clozapine overdose after a differential diagnosis. After gastric lavage and the first hemoperfusion, the blood clozapine concentration dropped to 1847.11 μg/L. However, this result was still higher than the laboratory alert concentration. So, the second hemoperfusion was administered, and then the second blood sample was sent to our laboratory to detect the real-time blood clozapine concentration.

Unexpectedly, the result significantly increased from 1847.11 μg/L to 5554.15 μg/L, exceeding the initial result. We scrutinized the laboratory data. Then, we immediately communicated with the clinical physician and knew that the patient’s clinical symptoms were not significantly better than before. She had a fever accompanied by sputum, and her arterial blood gas indicated hypoxemia. Several vital laboratory indicators were elevated, including inflammatory indexes and cardiac markers (Table 1). This abnormal blood clozapine concentration increase may be related to delayed absorption, and we recommend continuous monitoring of blood clozapine concentration. The patient was transferred to the intensive care unit based on those conditions. In order to accelerate the clearance of clozapine in the body, hemoperfusion was adjusted to twice a day. Fluid infusion and diuresis were continuously administered, and the patient’s blood clozapine concentration was monitored. The monitoring results are shown in Table 1. On Day 8 of the hospitalization, the blood clozapine concentration decreased to 1808.37 μg/L. Nevertheless, on the next day, a similar concentration fluctuation happened as before.

**Table 1:** Clinical and laboratory indicators during the patient’s hospitalization

**Indicators**	Day 0	D1	D2	D4	D5	D6	D7	D8	D9	D10	D11	D12	D13	D28
HR (bpm)	118	134	120	128	118	106	113	130	120	118	120	108	110	87
Pulse(rate/min)	126	126	126	118	104	108	110	116	108	120	118	104	118	82
BP (mmHg)	88/29	121/44	93/53	113/57	91/35	91/42	103/43	128/41	143/60	130/81	131/55	142/73	145/67	109/51
cTnI(ng/mL)	0.01	0.04	0.01	/	/	/	/	/	0.07	0.04	0.04	0.02	/	/
NT-ProBNP(pg/mL)	/	539	434	293	389	1100	1050	1030	18890	16300	12500	7970	/	566
WBC(× 10^9^/L)	12.98	14.61	20.46	17.14	14.49	11.07	13.77	11.82	18.32	13.51	12.2	14.46	19.24	/
PCT (ng/mL)	/	0.064	/	1.32	2.18	/	1.348	/	1.1	0.717	0.264	/	0.201	0.06
ALT(U/L)	/	31	23	43	43	42	39	/	66	41	/	31	/	/
AST(U/L)	/	26	35	48	41	49	45	/	92	37	/	35	/	/
GGT(U/L)	/	31	27	40	46	76	107	/	175	128	/	73	/	/
TBIL(μmol/L)	/	22.1	17.1	21.2	28.9	12.3	13.9	/	12.3	20.1	/	32	/	/
UREA (mmol/L)	/	2.69	1.9	4.55	10.22	6.99	6.4	6.95	10.5	14.32	16.04	14.1	/	/
CREA(μmol/L)	/	/	/	91	93	92	97	102	288	123	98	74	80	31
Clozapine(μg/L)	5200	1847.11	/	5554.15	4150.66	3389.82	2891.42	1808.37	2305.30	/	1084.58	/	495.46	26.36

**Fig. 1:**
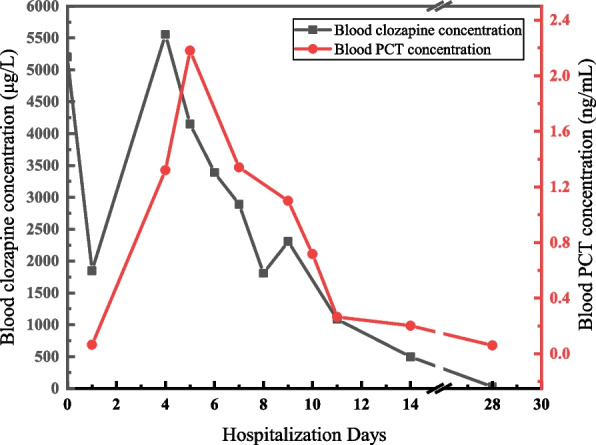
Blood clozapine concentration and PCT concentration of the patient during hospitalization

The blood clozapine concentration rose to 2303 μg/L, which increased by 27% compared with the result of Day 8. After reviewing medical records, we found that the clinical physician stopped the administration of hemoperfusion after Day 8, and several vital laboratory indicators (e.g., Creatinine, CREA; UREA; N-terminal Pro-Natriuretic Peptide, NT-ProBNP, γ-glutamyl transpetadase, γ-GGT) were raised in varying degrees. After communicating this abnormal blood concentration increase with the clinical physician, we recommend continuing hemoperfusion based on other necessary medical treatments and monitoring the blood levels two days later. Afterward, another hemoperfusion, anti-inflammatory, and anti-heart failure treatment were administered. On Day 11, the result decreased to 1084.58 μg/L, which decreased by 53% compared to Day 9 (Fig. 1). After 14 days of routine maintenance therapy, the patient’s blood clozapine concentration decreased to the therapeutic concentration range, and her mental state improved. She was transferred from the intensive care unit to the general ward to continue treatment until stabilized.

## Discussion

Generally, the routine oral dose of clozapine tablet is 25 mg/d, and the maximum dose is 600 mg/d. Some patients (e.g., rapid metabolizers, smokers) may require higher doses (e.g., 900 mg/d) to achieve therapeutic blood concentrations. Patients will likely experience adverse reactions when their blood concentration exceeds 600 μg/L [9]. According to pharmacokinetics, the oral absorption of clozapine is fast and complete, and it is distributed rapidly and widely to all tissues after absorption with high lipid solubility. For its bioavailability, the average individual variation was 50%-60% and it has a hepatic first-pass effect. The peak plasma concentration is reached 3.2 h (1-4 h) after dosing, the elimination half-life is 12–16 h on average, the apparent volume of distribution (Vd) is 4.04–13.78L/kg, and the protein binding rate is up to 94% [10]. Clozapine plasma levels show a 20-fold variability at the same dose (mg) taken [11]. It is mainly metabolized in the liver [12], and 80% excreted as metabolites in the urine and feces. Moreover, renal clearance and metabolism are significantly reduced in the elderly at the same dose with a specific body weight [13]. According to some reports, accidental overdose leading to death by poisoning has occurred occasionally [14]. To our knowledge, this case is the oldest one to date that has survived treatment after taking such a recorded high dose. By exploring the reference database, some reports lacked continuous blood concentration results [15], others didn’t accurately quantify the levels, especially the upper limit level [16].

Theoretically, after a series of treatments such as gastric lavage and hemoperfusion, the blood clozapine concentration should show a downward trend. In this case, however, we found the abnormal fluctuations (Fig. 1). After these abnormal blood concentration changes, we double-checked and investigated each batch’s sample pre-treatment, loading, quality control, and calibration curve in the testing process without any problems. In the meantime, we learned from the physicians that the clinical manifestation of the patients was consistent with the test results.

Several factors play roles in determining the blood clozapine concentration. Firstly, the patient was a female over 70, and the abnormal blood clozapine changes may be related to the decreased clearance due to age and sex [17]. Secondly, clozapine is metabolized primarily by human cytochrome P450 (CYP) isozyme 1A2 [18]. Systemic infection and inflammation may inhibit CYP450 enzymes, potentially leading to the risk of raised clozapine levels in people hospitalized for severe infections. Cytokines released in systemic inflammation with or without infection increase blood clozapine concentration by inhibiting the metabolism of CYP1A2 [19]. This outcome can be seen in Fig. 1. There was a rapid increase in blood procalcitonin (PCT) concentration on Day 4 and Day 5. The blood PCT concentration was still elevated on Day 9. Therefore, the patient’s systemic inflammation may have contributed to increased blood clozapine concentration. In addition, it is generally shown that the drug has been excreted to the small intestine after six hours of oral administration [20], but after taking large doses, many more clozapine tablets are embedded in the folds of the gastric mucosa for a more extended period and have not yet been excreted into the intestine [21], coupled with the fact that the tablet forms a block when it enters the stomach, making it difficult to dissolve and absorb. Timely gastric lavage and first hemoperfusion only clear metabolism partly in blood, which results in the decrease of blood clozapine concentration. For gastric motility and emptying were inhibited, metabolism accumulated continuously, which may significantly influence the re-increase in blood clozapine concentration.

Moreover, the use of hemoperfusion removes toxins from the blood in order to prevent the patient’s body from continuously taking up toxins and reduces the concentration of drugs in the blood [22]. Due to different sorbents, the clearance may be unsatisfactory, which can be improved by increasing the frequency of hemoperfusion [23]. On Day 8, however, judged by the decreased blood concentration, the patient did not receive another hemoperfusion, which may cause the re-increase of blood concentration.

To obtain good efficacy through individualized medication, TDM of clozapine is necessary as is highly recommended in 2017 AGNP guideline(Grade 1), especially for elderly patients who are complicated with chronic diseases and taking other drugs. Due to high sensitivity, accuracy, and excellent linear range, liquid chromatography-mass spectrometry is one of the best alternatives to monitor blood clozapine concentration [24]. Additionally, in the emergency poison screening, a comprehensive consideration of pharmacokinetics and factors directly or indirectly affecting blood concentration is crucial. In cases where laboratory results are inconsistent with the patient’s clinical symptoms, proactive communication with clinicians and a multifaceted exploration, including medical history, treatment plans, drug interactions, and other laboratory indicators, may adequately provide valuable guidance for further clinical treatment.

## Conclusions

Our case presents the highest reported blood clozapine level in an elderly woman (71 years old) who survived an overdose of 10,000 mg. This emphasizes the importance of clinical vigilance for unusual changes in blood drug concentrations following routine gastric lavage and hemoperfusion.

## Data Availability

Data sharing does not apply to this article, as this is a single-patient case report. No datasets besides those reported in the article were generated during the current study.

## Abbreviations

DA4R: Dopamine 4 receptor
TDM: Therapeutic drug monitoring
CYP450: Cytochrome P450
HR: Heart rate
BP: Blood pressure
ALT: Alanine transaminase
AST: Aspartate transaminase
TBIL: Total bilirubin
WBC: White blood cell
cTnI: Cardiac troponin I
PCT: Procalcitonin
CREA: Creatinine
NT-ProBNP: N-Terminal Pro-Natriuretic Peptide
γ-GGT: γ-Glutamyl transpetadase

## References

[1] Nucifora FC, Mihaljevic M, Lee BJ, Sawa A (2017). Clozapine as a model for antipsychotic development. Neurotherapeutics.

[2] Centorrino F, Baldessarini RJ, Kando JC, Frankenburg FR, Volpicelli SA, Flood JG (1994). Clozapine and metabolites: concentrations in serum and clinical findings during treatment of chronically psychotic patients. J Clin Psychopharmacol.

[3] Martin-Loeches I (2022). Therapeutic drug monitoring (TDM) in real-time: a need for the present future. Expert Rev Anti Infect Ther.

[4] Hiemke C, Bergemann N, Clement HW, Conca A, Deckert J, Domschke K (2018). Consensus guidelines for therapeutic drug monitoring in neuropsychopharmacology: update 2017. Pharmacopsychiatry.

[5] Jann MW (1991). Clozapine. Pharmacotherapy.

[6] Yada Y, Kitagawa K, Sakamoto S, Ozawa A, Nakada A, Kashiwagi H (2021). The relationship between plasma clozapine concentration and clinical outcome: a cross-sectional study. Acta Psychiatr Scand.

[7] Clark SR, Warren NS, Kim G, Jankowiak D, Schubert KO, Kisely S (2018). Elevated clozapine levels associated with infection: a systematic review. Schizophr Res.

[8] Ruan CJ, Zang YN, Wang CY, Cheng YH, Sun C, Spina E (2019). Clozapine metabolism in East Asians and Caucasians: a pilot exploration of the prevalence of poor metabolizers and a systematic review. J Clin Psychopharmacol.

[9] Rajkumar AP, Poonkuzhali B, Kuruvilla A, Jacob M, Jacob KS (2013). Clinical predictors of serum clozapine levels in patients with treatment-resistant schizophrenia. Int Clin Psychopharmacol.

[10] Telles-Correia D, Barbosa A, Cortez-Pinto H, Campos C, Rocha NBF, Machado S (2017). Psychotropic drugs and liver disease: a critical review of pharmacokinetics and liver toxicity. World J Gastrointest Pharmacol Ther.

[11] Mauri MC, Paletta S, Di Pace C, Reggiori A, Cirnigliaro G, Valli I (2018). Clinical pharmacokinetics of atypical antipsychotics: an update. Clin Pharmacokinet.

[12] Dias CL, Fonseca L, Gadelha A, Noto C (2021). Clozapine-induced hepatotoxicity: a life threatening situation. Schizophr Res.

[13] Manu P, Lapitskaya Y, Shaikh A, Nielsen J (2018). Clozapine rechallenge after major adverse effects: clinical guidelines based on 259 cases. Am J Ther.

[14] Krämer I, Rauber-Lüthy C, Kupferschmidt H, Krähenbühl S, Ceschi A (2010). Minimal dose for severe poisoning and influencing factors in acute human clozapine intoxication: a 13-year retrospective study. Clin Neuropharmacol.

[15] Sahyouni C, Hefazi E (2021). Clozapine induced pericarditis: a case report. Psychiatry Res.

[16] Bagade R, Karia S, Shah N, Andrade C (2022). Surviving a 10,000 mg overdose of clozapine: a case report. Schizophr Res.

[17] Ismail Z, Wessels AM, Uchida H, Ng W, Mamo DC, Rajji TK (2012). Age and sex impact clozapine plasma concentrations in inpatients and outpatients with schizophrenia. Am J Geriatr Psychiatry.

[18] Bertilsson L, Carrillo JA, Dahl ML, Llerena A, Alm C, Bondesson U (1994). Clozapine disposition covaries with CYP1A2 activity determined by a caffeine test. Br J Clin Pharmacol.

[19] de Leon J, Diaz FJ (2003). Serious respiratory infections can increase clozapine levels and contribute to side effects: a case report. Prog Neuropsychopharmacol Biol Psychiatry.

[20] Reith D, Monteleone JPR, Whyte IM, Ebelling W, Holford NHG, Carter GL (1998). Features and toxicokinetics of clozapine in overdose. Ther Drug Monit.

[21] Fitzsimons J, Berk M, Lambert T, Bourin M, Dodd S (2005). A review of clozapine safety. Expert Opin Drug Saf.

[22] Ricci Z, Romagnoli S, Reis T, Bellomo R, Ronco C (2022). Hemoperfusion in the intensive care unit. Intensive Care Med.

[23] Ronco C, Bellomo R (2022). Hemoperfusion: technical aspects and state of the art. Crit Care.

[24] Maublanc J, Dulaurent S, Morichon J, Lachâtre G, Gaulier JM (2015). Identification and quantification of 35 psychotropic drugs and metabolites in hair by LC-MS/MS: application in forensic toxicology. Int J Legal Med.

